# The characteristic of the complete chloroplast genome of *Lithocarpus konishii* (Fagaceae), a rare and endemic species in South China

**DOI:** 10.1080/23802359.2023.2226259

**Published:** 2023-06-21

**Authors:** Keyi Fu, Linjing Lu, Mingyan Ding, Zhilai Yang, Hongkang Shen, Shi Shi

**Affiliations:** a Guangdong Key Laboratory for Innovative Development and Utilization of Forest Plant Germplasm, South China Agricultural University, Guangzhou, China; b South China Limestone Plants Research Center, College of Forestry and Landscape Architecture, South China Agricultural University, Guangzhou, China; c Shunde Polytechnic, Foshan, China

**Keywords:** *Lithocarpus konishii*, chloroplast genome, next-generation sequencing, phylogenetic relationships analysis

## Abstract

*Lithocarpus konishii*, a rare species endemic to islands in South China, was evaluated as a vulnerable species (VU) by the ‘China Species Red List.’ Here, we first presented the complete chloroplast genome sequence of *L. konishii.* The chloroplast genome was 161,059 bp in length with 36.76% GC content, containing a small single-copy region (SSC, 18,967 bp), a large single-copy region (LSC, 90,250 bp), and a pair of inverted repeats (IRs, 25,921 bp each). A total of 139 genes were predicted, including 87 protein-coding genes (CDS), 8 rRNAs, and 44 tRNAs. Based on the concatenated shared unique CDS sequence dataset, maximum-likelihood and Bayesian inference methods were used to build the phylogenetic trees of 18 species from the Fagaceae family. Results indicated that *L. konishii* is closely related to *L. longnux* and *L. pachyphyllus* var. *fruticosus*, and forms a monophyly of the subfamily Castaneoideae with *Castanopsis* and *Castanea*. This study provides a theoretical basis for the conservation genomics of this endangered plant.

## Introduction

**Figure 1.**
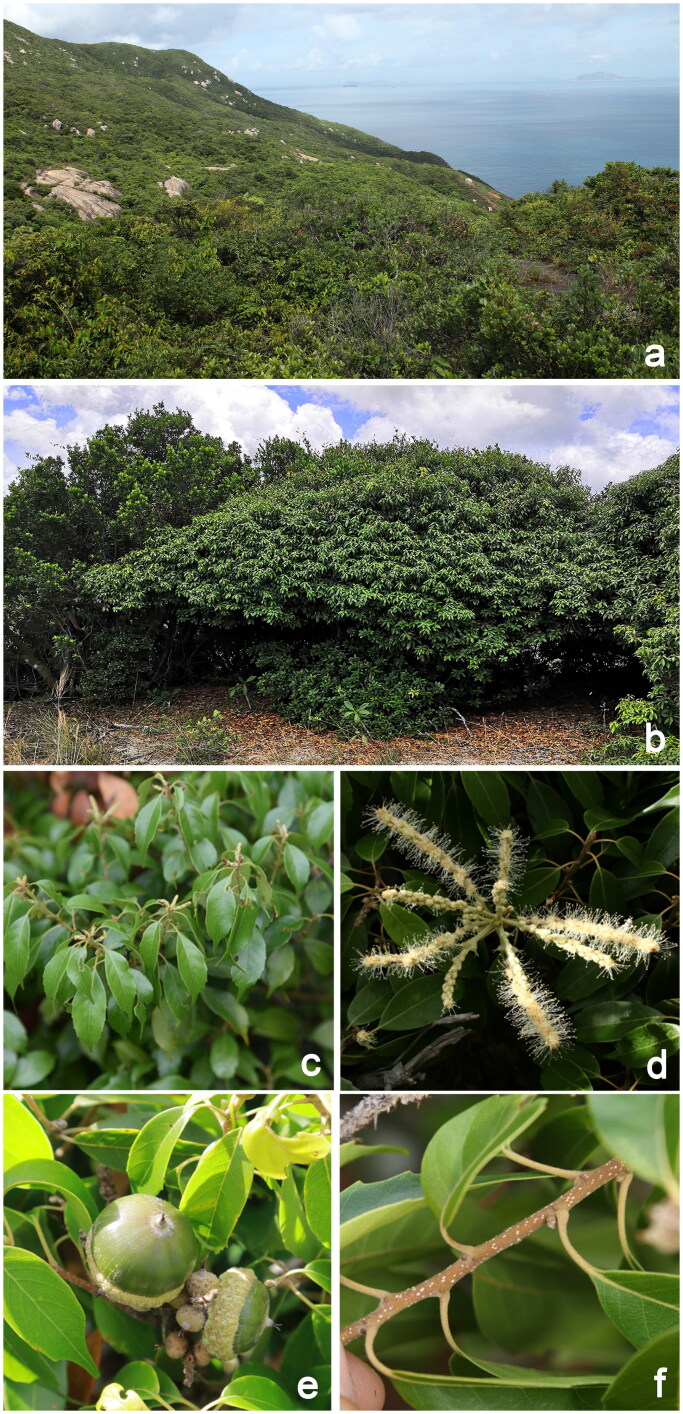
*Lithocarpus konishii* (a) Natural habitat; (b) the entire plant; (c) leaf; (d) flower; (e) fruit; and (f) twig. The images were taken by Shi Shi on Dangan Island, Guangdong Province (114°14'48.89" E, 22°02'13.43" N).

*Lithocarpus konishii* (Hayata) Hayata 1917 is a small evergreen tree belonging to the Fagaceae family, and is endemic to South China (Huang et al. 1999). It is found sporadically in a few island habitats in Hainan, Zhuhai, Hong Kong, and Taiwan, indicating a typical island disjunctive distribution (Shi et al. 2016). *Lithocarpus konishii* typically grows between 4 and 9 meters in height, with papery leaf blade, acute to caudate-acuminate apex, 3–6 obtuse teeth leaf margin, depressed globose nut, and discoid cupule (Figure 1). It blossoms twice a year in April and August, with fruits ripening from July to October (Huang et al. 1999; Hung et al. 2005). *L. konishii* exhibits exceptional resilience to salt, alkali, drought, and sterile conditions. Additionally, it has robust wind resistance capabilities and plays a vital role in preserving water and soil within island ecosystems (Shi et al. 2016). Furthermore, *L. konishii* holds significant economic value and shows promising potential for development. For instance, the fruits are consumed by residents in Hainan after cooking, while in Taiwan, it has been cultivated as a landscaping tree and a source for truffle reproduction (You 2021).

However, habitat destruction and excessive deforestation have resulted in grave habitat fragmentation, population decline, and wild germplasm resources reduction of *L. konishii*. Based on a study utilizing chloroplast DNA atpB-rbcL for the genetic diversity analysis of *L. konishii* in Taiwan, it was found that the habitat of this species was severely damaged by the 1999 Chi-Chi earthquake, which resulted in a substantial loss of genetic diversity, placing *L. konishii* on the brink of endangerment in Taiwan. (Hung et al. 2005). Moreover, in the eastern part of Hainan, the population of *L. konishii* has been strongly affected by human activities. The expansion of roads, farmland, and housing have directly encroached upon its habitat (Shi et al. 2016). Due to the scarcity of wild populations of *L. konishii*, it has been evaluated as a vulnerable species (VU) and listed in the 'China Species Red List’ (Wang and Xie 2004). However, few studies have focused on the maintenance and conservation of this species. This study sequenced the complete chloroplast genome of *L. konishii*, and explored its phylogenetic relationships with other species in the Fagaceae family, which could be valuable to the effective utilization and protection of this species, as well as the further phylogenetic studies of this family.

## Materials

Fresh and young leaves of *L. konishii* were collected from Dangan Island, Guangdong Province, China (114°14′48.89″ E, 22°02′13.43″ N). The voucher specimen of the sample was deposited at the Herbarium of South China Agricultural University (CANT, https://nbb.scau.edu.cn/) under the accession number 32206 (contact person: Mingxuan Zheng, zhengmx@scau.edu.cn).

## Methods

**Figure 2.**
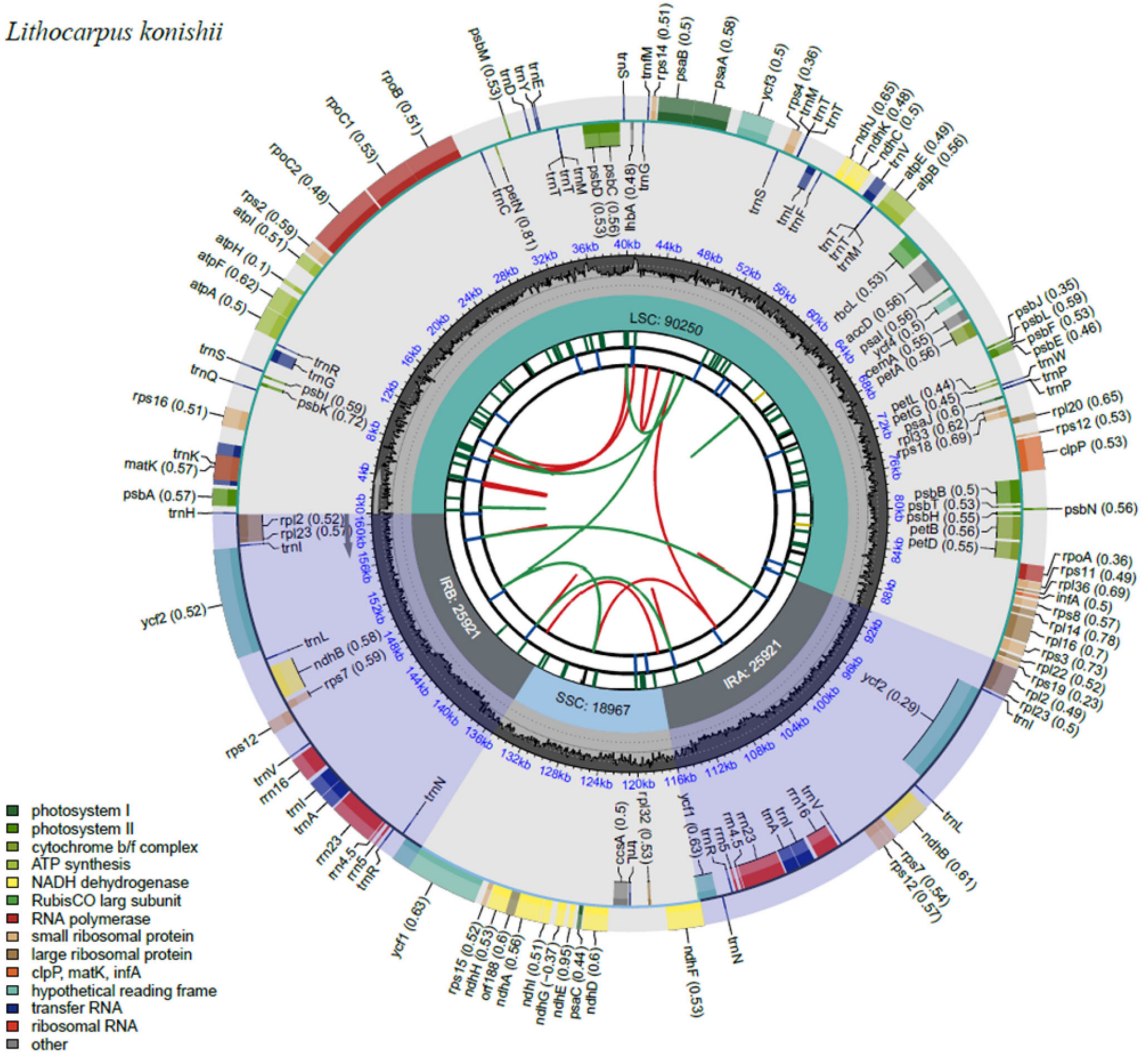
Genome map of *Lithocarpus konishii.* The map contains six tracks by default. From the center outward, the first track shows the dispersed repeats, which consist of direct (D) and palindromic (P) repeats, connected with red and green arcs. The second track shows the long tandem repeats as short blue bars. The third track shows the short tandem repeats or microsatellite sequences as short bars with different colors. The colors, the type of repeat they represent, and the description of the repeat types are as follows. Black: c (complex repeat); green: p1 (repeat unit size = 1); yellow: p2 (repeat unit size = 2); purple: p3 (repeat unit size = 3); blue: p4 (repeat unit size = 4); orange: p5 (repeat unit size = 5); red: p6 (repeat unit size = 6). The small single-copy (SSC), inverted repeat (IRa and IRb), and large single-copy (LSC) regions are shown on the fourth track. The GC content along the genome is plotted on the fifth track. The genes are shown on the sixth track. The optional codon usage bias is displayed in the parenthesis after the gene name. Genes are color-coded by their functional classification. The transcription directions for the inner and outer genes are clockwise and anticlockwise, respectively. The bottom left corner shows the functional classifications of the genes.

The total genomic DNA was extracted from fresh leaves using a modified cetyl trimethyl ammonium bromide (CTAB) method (Doyle and Doyle 1987). A genomic library consisting of an insert size of 300 bp was established by using a TruSeq DNA Sample Prep Kit (Illumina, USA) and sequenced on the Illumina Novaseq platform (Guangzhou Jierui Biotech). 5 Gb of raw data of 150 bp paired-end reads were obtained and further assembled using GetOrganelle v.1.7.7.0 (Jin et al. 2020). The GeSeq was used for chloroplast genome annotation (Tillich et al. 2017), whilst CPGAVAS2 was used to correct the annotated genome (Shi et al. 2019), after which it was manually checked by comparison against the complete cp genome of *Lithocarpus hancei* (GenBank accession number: MW375417) using Geneious v.9.0.2 (Biomatters, https://www.geneious.com) (Kearse et al. 2012). The complete chloroplast genome of *L. konishii* was submitted to GenBank with the accession number ON422319. The chloroplast Genome Viewer (CPGView, www.1kmpg.cn/cpgview/) (Liu et al. 2023) was used to visualize the structural features of *L. konishii* (Figure 2).

To further understand the intrageneric phylogenetic relationship of *L. konishii*, the complete chloroplast genome sequences of 18 species of family Fagaceae and 2 outgroup species (*Morella salicifolia* and *Carpinus laxiflora*) from the National Center for Biotechnology Information (NCBI) were aligned using MUSCLE v.3.8.31 (Edgar 2004). Based on the concatenated shared unique CDS sequence dataset, the phylogenetic trees were constructed using the maximum likelihood (ML) method by IQ-TREE v.2.0.3 (Nguyen et al. 2015) and Bayesian Inference (BI) method by Mrbayes v.3.2.6 (Ronquist et al. 2012). For ML analysis, a best-fit model K3Pu + F + I was selected, and the reliability of the phylogenetic tree topology was evaluated with 1,000 repeated self-expanding analyses; while for BI analysis, a best-fit model GTR + G + I was estimated by ModelTest-NG v.0.1.7 (Darriba et al. 2020) on the CIPRES Science Gateway (http://www.phylo.org/portal2/), the Markov Chain Monte Carlo (MCMC) was conducted for 5,000,000 generations and sampled every 100 iterations with the first 20% discarded. Branch supports were tested using the ultrafast bootstrap (UFBoot) (Hoang et al. 2018).

## Results and discussion

The minimum and average coverage of the assembled chloroplast genome were 97× and 345.71×, respectively (Supplementary Figure 1). The complete chloroplast genome of *L. konishii* (ON422319) was 161,059 bp in length, presenting a typical quadripartite structure containing a small single-copy region (SSC,18,967 bp) and a large single-copy region (LSC, 90,250 bp), separated by a pair of inverted repeats (IRs, 25,921 bp each). The overall GC content of the cp genome was 36.76%. The cp genome encoded 139 genes, including 87 protein-coding genes (CDS), 8 rRNA genes, and 44 tRNA genes. There were 19 genes (*rps16, atpF, rpoC1, petB, petD, rp116, rp12, ndhB, ndhA, ndhB_copy2, rp12_copy2, trnK-UUU, trnG-GCC, trnL-UAA, trnV-UAC, trnI-GAU, trnA-UGC, trnA-UGC_copy2, trnI-GAU_copy2*) containing 1 intron and 4 genes (*rps12, ycf3, rps12_copy2, clpP*) containing 2 introns (Supplementary Figure 2), while the gene structure of the trans-splicing gene rps12 was identified, which has 3 unique exons, 2 of them are duplicated as they are located in the IR regions (Supplementary Figure 3).

**Figure 3.**
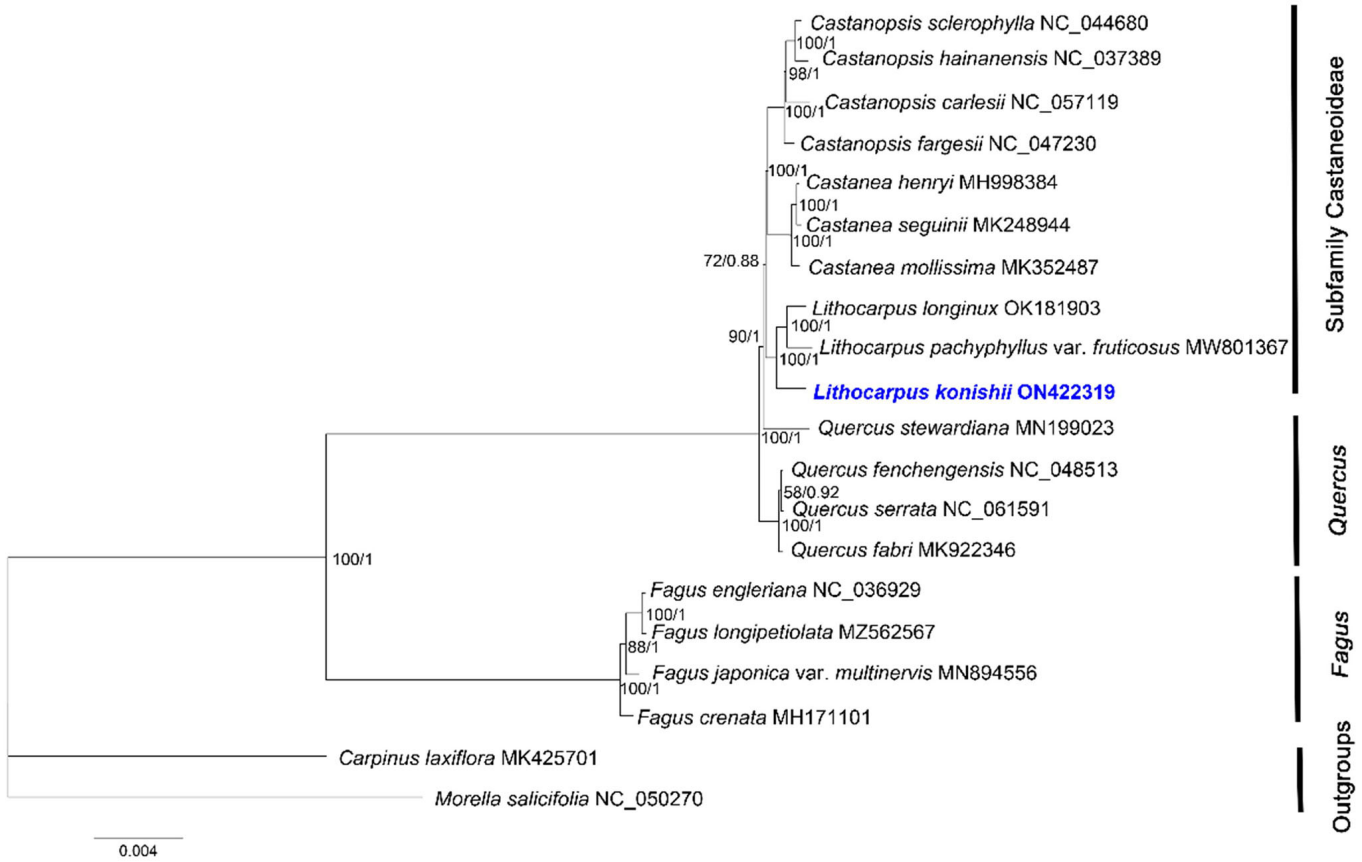
The phylogenetic tree based on the complete chloroplast genome sequences of 18 species from the Fagaceae family, with *Morella salicifolia* and *Carpinus laxiflora* as Outgroups. The following sequences were used: *Castanopsis sclerophylla* NC_044680 (Ye, Hu, et al. 2019), *Castanopsis hainanensis* NC_037389 (Chen et al. 2018), *Castanopsis carlesii* NC_057119 (Sun et al. 2019), *Castanopsis fargesii* NC_047230 (Ye, Guo, et al. 2019), *Castanea henryi* MH998384 (Gao et al. 2019), *Castanea seguinii* MK248944 (Chen et al. 2019), *Castanea mollissima* MK352487 (Zhu et al. 2019), *Lithocarpus longinux* OK181903 (Wu et al. 2022), *Lithocarpus pachyphyllus* var. *fruticosus* MW801367 (Jin et al. 2021), *Quercus stewardiana* MN199023 (Li et al. 2020), *Quercus fenchengensis* NC_048513 (Hu et al. 2019), *Quercus serrata* NC_061591 (Yang et al. 2021), *Quercus fabri* MK922346 (Xu et al. 2019), *Fagus engleriana* NC_036929 (Yang et al. 2018), *Fagus longipetiolata* MZ562567 (Liang et al. 2022), *Fagus japonica* var. *multinervis* MN894556 (Park and Oh 2020), *Fagus crenata* MH171101 (Worth et al. 2019), *Carpinus laxiflora* MK425701 (Lee et al. 2019), and *Morella salicifolia* NC_050270 (Karumuna et al. 2019). NCBI accession numbers of each genome are shown in the figure. The bootstrap support values were based on 1000 replicates. Values of bootstrap support and posterior probability for each branch nodes are as indicated.

Both the ML and BI trees displayed identical topologies, here we presented the ML tree (Figure 3). The phylogenetic analysis shows that *L. konishii* is closely related to *L. longnux* and *L. pachyphyllus* var. *fruticosus*, and is the sister clade of *Castanopsis* and *Castanea*, forming a monophyly of the subfamily Castaneoideae, in agreement with the findings based on the nuclear and chloroplast DNA sequence data by Manos et al. (2001) and the morphological data by Wang and Bo (2004). *Fagus* represented an early branch within the family, consistent with the previous phylogeny studies of Fagaceae (Manos et al. 2001; Yan 2021; Wang and Bo 2004). These findings can serve as a valuable chloroplast genome resource for genetic research on germplasm resources update and afforestation tree species in the future.

## Supplementary Material

Supplemental Material

Supplemental Material

Supplemental Material

## Data Availability

The data that support the findings of this study are openly available in NCBI at [https://www.ncbi.nlm.nih.gov/], reference number ON422319. The associated “BioProject”, “Bio-Sample” and “SRA” numbers are PRJNA838448, SAMN28422863 and SRR19233775 respectively.

## References

[CIT0002] Chen X, Liu QZ, Guo W, Xu L, Zhang LS. 2019. The complete chloroplast genome of *Castanea seguinii*, an endemic to China. Mitochondrial DNA Part B. 4(1):758–759. doi: 10.1080/23802359.2019.1565970.

[CIT0003] Chen XD, Yang J, Yang YC, Zhang X, Du XM, Zhao GF. 2018. Characterization of the complete plastid genome of *Castanopsis hainanensis* Merrill. Conservation Genet Resour. 10(4):825–828. doi: 10.1007/s12686-017-0940-9.

[CIT0004] Darriba D, Posada D, Kozlov AM, Stamatakis A, Morel B, Flouri T. 2020. ModelTest-NG: a new and scalable tool for the selection of DNA and protein evolutionary models. Mol Biol Evol. 37(1):291–294. doi: 10.1093/molbev/msz189. 31432070 PMC6984357

[CIT0005] Doyle JJ, Doyle JL. 1987. A rapid DNA isolation procedure for small quantities of fresh leaf tissue. Phytochem Bull. 19:11–15.

[CIT0006] Edgar RC. 2004. Muscle: multiple sequence alignment with high accuracy and high throughput. Nucleic Acids Res. 32(5):1792–1797. doi: 10.1093/nar/gkh340. 15034147 PMC390337

[CIT0007] Gao XX, Yan F, Liu M, Zulfiqar S, Zhao P. 2019. The complete chloroplast genome sequence of an endemic species Pearl chestnut (*Castanea henryi*). Mitochondrial DNA Part B. 4(1):551–552. doi: 10.1080/23802359.2018.1553522.

[CIT0008] Hoang DT, Chernomor O, von Haeseler A, Minh BQ, Vinh LS. 2018. UFBoot2: improving the ultrafast bootstrap approximation. Mol Biol Evol. 35(2):518–522. doi: 10.1093/molbev/msx281. 29077904 PMC5850222

[CIT0009] Hu HL, Wang LZ, Yang J, Zhang RS, Li Q, Liu YQ, Qin L. 2019. The complete chloroplast genome of *Quercus fenchengensis* and the phylogenetic implication. Mitochondrial DNA B Resour. 4(2):3066–3067. doi: 10.1080/23802359.2019.1666040. 33365858 PMC7706804

[CIT0010] Huang CJ, Zhang YT, Bartholo B. 1999. Flora of China. Vol. 4. Science Press; p. 358.

[CIT0011] Hung KH, Hsu TW, Schaal BA, Chiang TY. 2005. Loss of genetic diversity and erroneous phylogeographical inferences in *Lithocarpus konishii* (Fagaceae) of Taiwan caused by the Chi-Chi earthquake: implications for conservation. Ann Missouri Botan Garden. 92(1):52–65. DOI: http://www.jstor.org/stable/3298648.

[CIT0012] Jin JJ, Yu WB, Yang JB, Song Y, De Pamphilis CW, Yi TS, Li DZ. 2020. GetOrganelle: a fast and versatile toolkit for accurate de novo assembly of organelle genomes. Genome Biol. 21(1):1–31. doi: 10.1186/s13059-020-02154-5. PMC7488116 32912315

[CIT0013] Jin L, Liu JJ, Xiao TW, Li QM, Lin LX, Shao XN, Ma CX, Li BH, Mi XC, Qiao XJ, et al. 2021. Community phylogenetics require phylogenies reconstructed from plastid genomes. Authorea Preprints. doi: 10.22541/au.161834751.14170237/v1.

[CIT0014] Karumuna JJ, Yan DY, Kyalo CM, Li ZZ. 2019. The complete chloroplast genome sequence of *Morella salicifolia* (Myricaceae): characterization and phylogenetic analysis. Mitochondrial DNA Part B. 4(1):963–964. doi: 10.1080/23802359.2019.1580157.

[CIT0015] Kearse M, Moir R, Wilson A, Stones-Havas S, Cheung M, Sturrock S, Buxton S, Cooper A, Markowitz S, Duran C, et al. 2012. Geneious Basic: an integrated and extendable desktop software platform for the organization and analysis of sequence data. Bioinformatics. 28(12):1647–1649. doi: 10.1093/bioinformatics/bts199. 22543367 PMC3371832

[CIT0016] Lee MW, Kim SC, Ahn JY, Lee JW. 2019. The complete chloroplast genome of *Carpinus Laxiflora* (Betulaceae). Mitochondrial DNA Part B. 4(1):1643–1644. doi: 10.1080/23802359.2019.1604184.

[CIT0017] Li Y, Wang L, Zhao Y, Fang YM. 2020. The complete chloroplast genome sequence of *Quercus stewardiana* (Fagaceae). Mitochondrial DNA Part B. 5(2):1958–1959. doi: 10.1080/23802359.2020.1756961.

[CIT1018] Liang DQ, Wang HY, Zhang J, Zhao YX, Wu F. 2022. Complete chloroplast genome sequence of Fagus longipetiolata Seemen (Fagaceae): Genome structure, adaptive evolution, and phylogenetic relationships. Life. 12(1):92. doi: 10.3390/life12010092. PMC8778281 35054485

[CIT0018] Liu SY, Ni Y, Li JL, Zhang XY, Yang HY, Chen HM, Liu C. 2023. CPGView: a package for visualizing detailed chloroplast genome structures. Mol Ecol Resour. 23(3):694–704. doi: 10.1111/1755-0998.13729. 36587992

[CIT0019] Manos PS, Zhou ZK, Cannon CH. 2001. Systematics of Fagaceae: phylogenetic tests of reproductive trait evolution. Int J Plant Sci. 162(6):1361–1379. doi: 10.1086/322949.

[CIT0020] Nguyen LT, Schmidt HA, Arndt VH, Quang MB. 2015. IQ-TREE: a fast and effective stochastic algorithm for estimating maximum-likelihood phylogenies. Mol Biol Evol. 32(1):268–274. doi: 10.1093/molbev/msu300. 25371430 PMC4271533

[CIT0021] Park JS, Oh SH. 2020. A second complete chloroplast genome sequence of *Fagus multinervis* Nakai (Fagaceae): intraspecific variations on chloroplast genome. Mitochondrial DNA Part B. 5(2):1868–1869. doi: 10.1080/23802359.2020.1752837.

[CIT0022] Ronquist F, Teslenko M, van der Mark P, Ayres DL, Darling A, Höhna S, Larget B, Liu L, Suchard MA, Huelsenbeck JP. 2012. Mrbayes 3.2: efficient Bayesian phylogenetic inference and model choice across a large model space. System Biol. 61(3):539–542. doi: 10.1093/sysbio/sys029. 22357727 PMC3329765

[CIT0023] Shi LC, Chen HM, Jiang M, Wang LQ, Wu X, Huang LF, Liu C. 2019. CPGAVAS2, an integrated plastome sequence annotator and analyzer. Nucleic Acids Res. 47(W1):W65–W73. doi: 10.1093/nar/gkz345. 31066451 PMC6602467

[CIT0024] Shi S, Fan Q, Chen SF, Tan WZ. 2016. Study on the biogeographic pattern of the discontinuous distribution between Hainan and Taiwan – based on the genetic geography analysis of Coleoptera. Beijing: China Science and Technology (paper online)

[CIT0025] Sun RX, Ye XM, Wang ZL, Lin XF. 2019. The complete chloroplast genome of *Castanopsis carlesii* (Hemsl.) Hay. Mitochondrial DNA B Resour. 4(2):2591–2592. doi: 10.1080/23802359.2019.1641437. 33365639 PMC7706500

[CIT0026] Tillich M, Lehwark P, Pellizzer T, Ulbricht-Jones ES, Fischer A, Bock R, Greiner S. 2017. GeSeq – versatile and accurate annotation of organelle genomes. Nucleic Acids Res. 45(W1):W6–W11. doi: 10.1093/nar/gkx391. 28486635 PMC5570176

[CIT0027] Wang PL, Bo FD. 2004. Pollen morphology and biogeography of Fagaceae plants. Guangzhou: Guangdong Science and Technology Press. p. 7–107.

[CIT0028] Wang S, Xie Y. 2004. China species red list: vol. I red list. Beijing: Higher Education Press. p. 314–315.

[CIT0029] Worth JRP, Liu L, Wei FJ, Tomaru N. 2019. The complete chloroplast genome of *Fagus crenata* (subgenus *Fagus*) and comparison with *F.engleriana* (subgenus *Engleriana*). PeerJ. 7:e7026. doi: 10.7717/peerj.7026. 31211014 PMC6557254

[CIT0030] Wu CY, Lin L, Yao KP, Yang RJ, Deng M. 2022. The complete chloroplast genome sequence of *Lithocarpus longinux* (Fagaceae). Mitochondrial DNA B Resour. 7(7):1229–1231. doi: 10.1080/23802359.2022.2093664. 35814181 PMC9262355

[CIT0031] Xu Y, Chen H, Qi M, Su W, Zhang Y, Du FK. 2019. The complete chloroplast genome of *Quercus fabri* (Fagaceae) from China. Mitochondrial DNA B Resour. 4(2):2857–2858. doi: 10.1080/23802359.2019.1660921. 33365761 PMC7706987

[CIT0032] Yan ZT. 2021. Research progress of *Fagus* plants in China. Agric Develop Equip. 240(12):107–108.

[CIT0033] Yang YC, Zhou T, Qian ZQ, Zhao GF. 2021. Phylogenetic relationships in Chinese oaks (Fagaceae, *Quercus*): Evidence from plastid genome using low-coverage whole genome sequencing. Genomics. 113(3):1438–1447. doi: 10.1016/j.ygeno.2021.03.013. 33744343

[CIT0034] Yang YC, Zhu J, Feng L, Zhou T, Bai GQ, Yang J, Zhao GF. 2018. Plastid genome comparative and phylogenetic analyses of the key genera in Fagaceae: highlighting the effect of codon composition bias in phylogenetic inference. Front Plant Sci. 9:82. doi: 10.3389/fpls.2018.00082. 29449857 PMC5800003

[CIT0035] Ye XM, Guo YP, Lei XG, Sun RX. 2019a. The complete chloroplast genome of *Castanopsis Fargesii* Franch. (Fagaceae). Mitochondrial DNA Part B. 4(1):1656–1657. doi: 10.1080/23802359.2019.1605850.

[CIT0036] Ye XM, Hu DG, Guo YP, Sun RX. 2019b. Complete chloroplast genome of *Castanopsis sclerophylla* (Lindl.) Schott: genome structure and comparative and phylogenetic analysis. PLOS One. 14(7):e0212325. doi: 10.1371/journal.pone.0212325. 31361757 PMC6667119

[CIT0037] You SF. 2021. The new species *Lithocarpus konishii* truffle published by Taiwan Forestry Research Institute. Agriculture Media [accessed 2021 Mar 30]. https://www.agriharvest.tw/archives/57060.

[CIT0039] Zhu CC, Shi FH, Wang M, Zhao YQ, Chen Y, Geng GM. 2019. The complete chloroplast genome of a variety of *Castanea mollissima* 'Hongli’ (Fagaceae). Mitochondrial DNA Part B. 4(1):993–994. doi: 10.1080/23802359.2019.1580160.

