# Supplementary figures and images for: The characteristic of the complete chloroplast genome of Lithocarpus konishii (Fagaceae), a rare and endemic species in South China
Source: Mitochondrial DNA B Resour. 2023 Jun 21;8(6):686–90. doi: 10.1080/23802359.2023.2226259 (PMC10286688; doi:10.1080/23802359.2023.2226259)

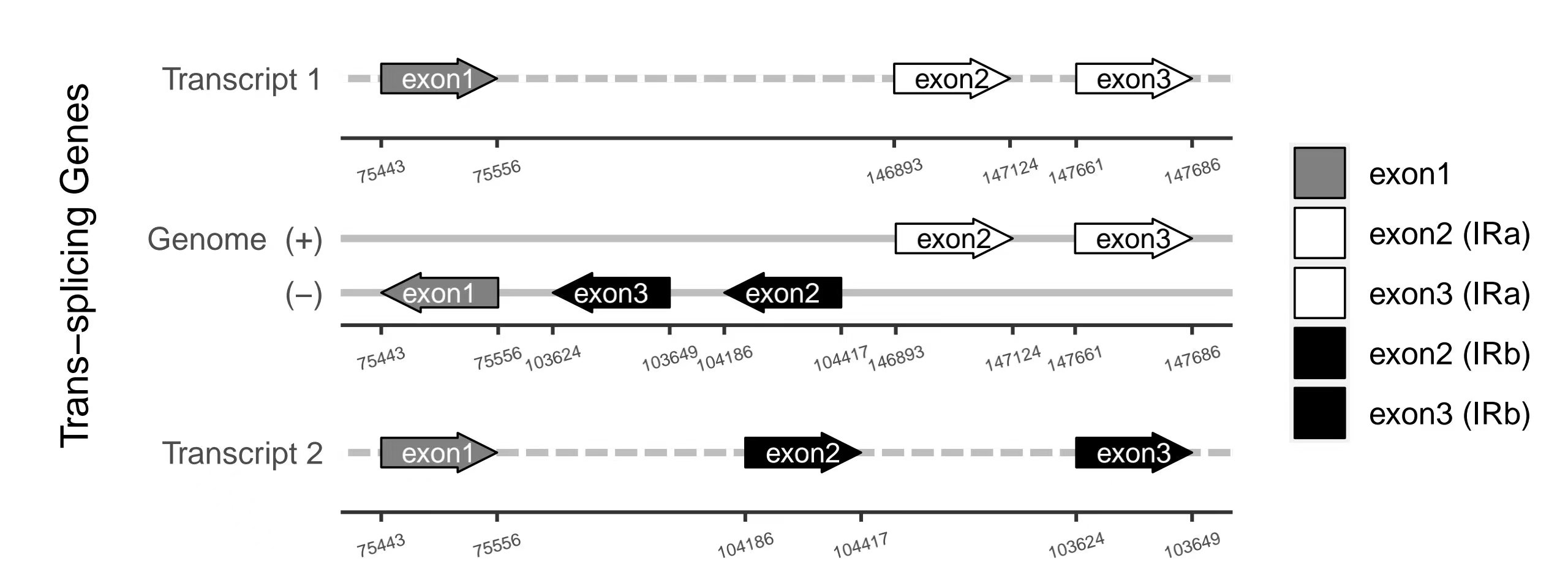

Supplement: Supplemental Material [file TMDN_A_2226259_SM1021.jpg]

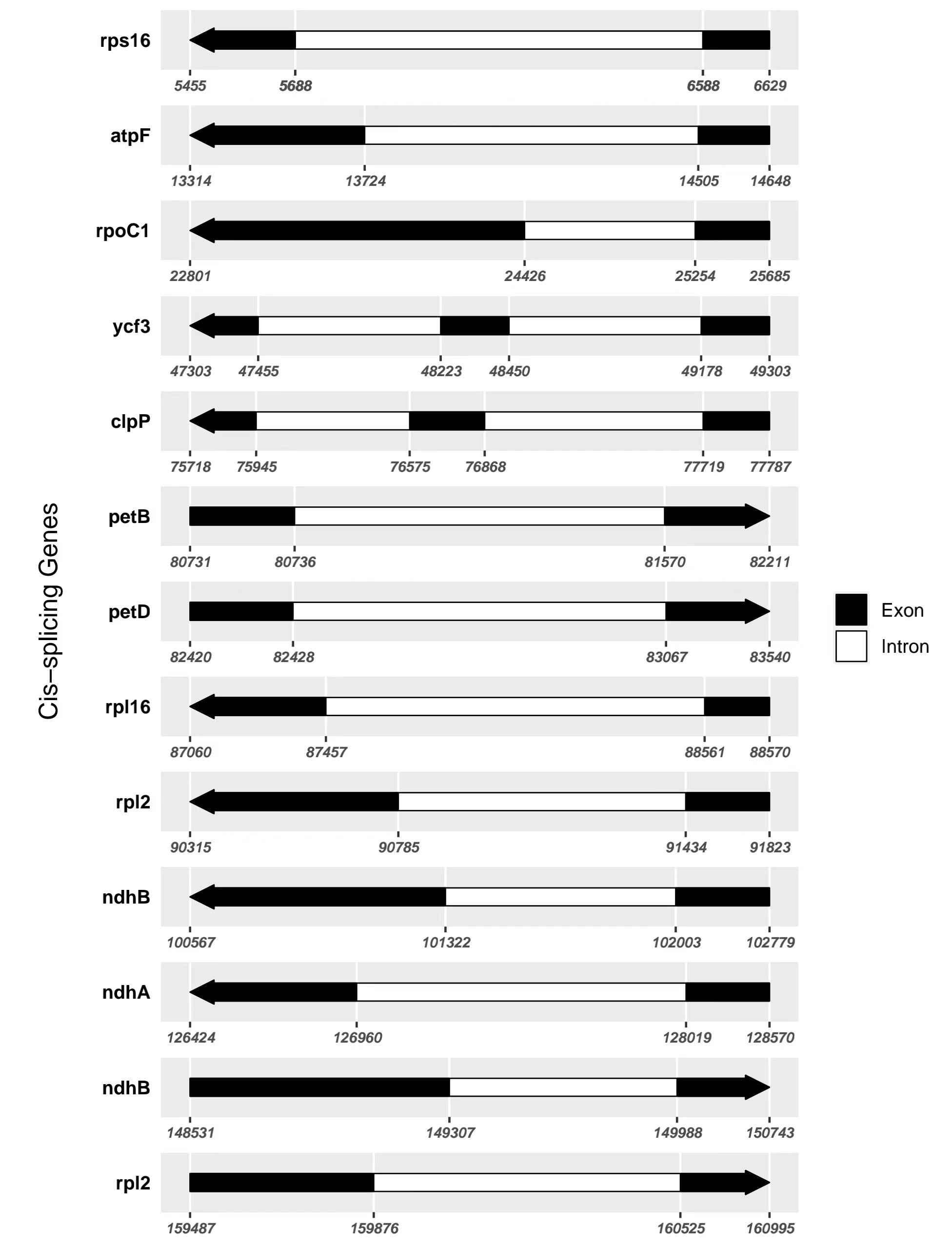

Supplement: Supplemental Material [file TMDN_A_2226259_SM1017.png]

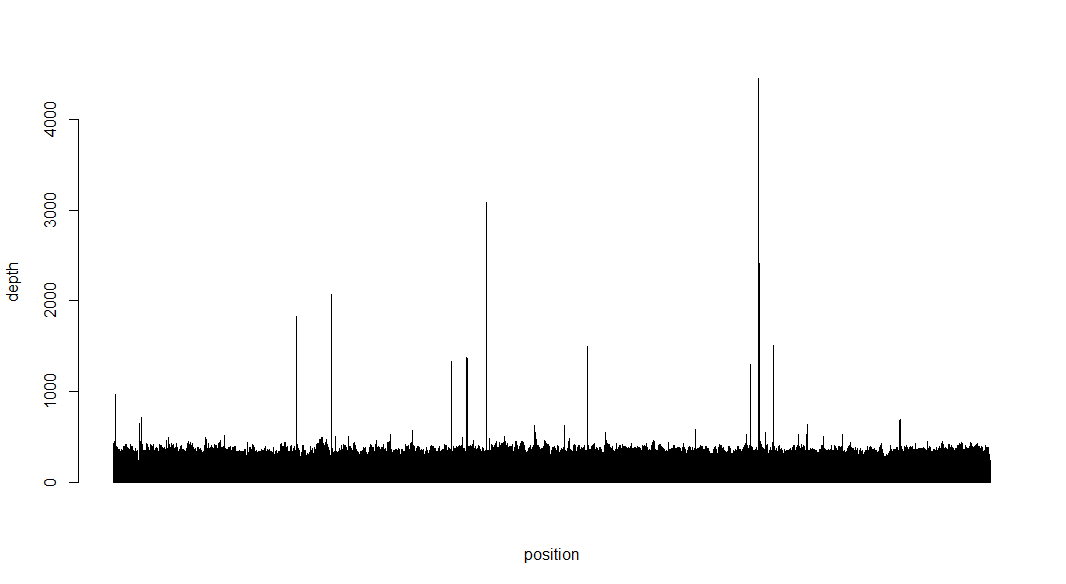

Supplement: Supplemental Material [file TMDN_A_2226259_SM1007.tiff]
